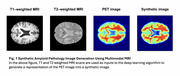# AI‐driven Amyloid Pathology Detection Using Multi‐modal MRI: A Proof of Concept Study

**DOI:** 10.1002/alz70856_099593

**Published:** 2025-12-24

**Authors:** Manmohi D Dake, Himank Kavathekar, Ashwin V Venkataraman, Dag Aarsland

**Affiliations:** ^1^ Silver Matter, Pune, Maharashtra, India; ^2^ Centre for Neuroimaging Sciences, IoPPN, King's College London, London, United Kingdom; ^3^ South London and Maudsley NHS Foundation Trust, London, United Kingdom; ^4^ Centre for Healthy Brain Ageing, IoPPN, King's College London, London, London, United Kingdom; ^5^ Institute of Psychiatry, Psychology and Neuroscience, King's College London, London, United Kingdom; ^6^ Centre for Age‐Related Medicine, Stavanger University Hospital, Stavanger, Stavanger, Norway

## Abstract

**Background:**

Traditional methods for detecting Alzheimer's disease (AD) pathology are often expensive, invasive, and inaccessible. Early identification of AD biomarkers is critical to enable timely and effective interventions that improve patient outcomes. To address these limitations, we developed a method to detect amyloid pathology, a hallmark of AD, using multi‐modal MRI—a cost‐effective, accessible, and non‐invasive imaging approach compared to traditional detection methods.

**Method:**

A deep learning model was trained using 1,488 paired AV‐45 amyloid PET and MRI scans from the Alzheimer's Disease Neuroimaging Initiative (ADNI), encompassing participants across the AD spectrum. The dataset was split into training, testing, and validation subsets (80:10:10). The model was validated on 149 participants across the AD continuum and cognitively healthy adults. T1‐weighted and T2‐weighted MRI scans were provided as inputs to synthesize an image representative of amyloid pathology (Figure 1). Standard Uptake Value Ratio (SUVR) scores were calculated for both the original PET images (ground truth) and the synthesized images. Sensitivity, specificity, and accuracy were assessed using an SUVR threshold of 1.55 to evaluate the model's ability to detect amyloid positivity.

**Result:**

On evaluation using the validation dataset, the model achieved a sensitivity of 0.70, specificity of 0.72, and accuracy of 0.71 in detecting amyloid positivity. The synthesized images demonstrated strong alignment with the original PET images, as reflected by similar SUVR scores and clinically relevant accuracy metrics.

**Conclusion:**

This proof of concept work provides a preliminary method to make amyloid detection safer, more affordable, and more accessible for patients and the healthcare industry. The technology leverages routinely acquired MRI scans to extract additional clinical insights, offering a non‐invasive tool for identifying candidates eligible for early disease‐modifying therapies. While further improvements to the model accuracy are ongoing, this approach has the potential to improve early detection and patient prognosis.